# The Fifth Bioelectronic Medicine Summit: today’s tools, tomorrow’s therapies

**DOI:** 10.1186/s42234-023-00123-4

**Published:** 2023-10-05

**Authors:** Eric H. Chang, Arielle H. Gabalski, Tomas S. Huerta, Timir Datta-Chaudhuri, Theodoros P. Zanos, Stavros Zanos, Warren M. Grill, Kevin J. Tracey, Yousef Al-Abed

**Affiliations:** 1 Feinstein Institutes for Medical Research, Northwell Health, 350 Community Drive, Manhasset, NY 11030 USA; 2 Donald and Barbara Zucker School of Medicine at Hofstra/Northwell, 500 Hofstra Blvd, Hempstead, NY 11549 USA; 3 The Elmezzi Graduate School of Molecular Medicine, Northwell Health, 350 Community Drive, Manhasset, NY 11030 USA; 4 Department of Biomedical Engineering, Fitzpatrick CIEMAS, Duke University, Room 1427, 101 Science Drive, Box 90281, Durham, NC 27708 USA

**Keywords:** Neurotechnology, Materials science, Preclinical research, Neuroimmunology, Neuromodulation

## Abstract

The emerging field of bioelectronic medicine (BEM) is poised to make a significant impact on the treatment of several neurological and inflammatory disorders. With several BEM therapies being recently approved for clinical use and others in late-phase clinical trials, the 2022 BEM summit was a timely scientific meeting convening a wide range of experts to discuss the latest developments in the field. The BEM Summit was held over two days in New York with more than thirty-five invited speakers and panelists comprised of researchers and experts from both academia and industry. The goal of the meeting was to bring international leaders together to discuss advances and cultivate collaborations in this emerging field that incorporates aspects of neuroscience, physiology, molecular medicine, engineering, and technology. This Meeting Report recaps the latest findings discussed at the Meeting and summarizes the main developments in this rapidly advancing interdisciplinary field. Our hope is that this Meeting Report will encourage researchers from academia and industry to push the field forward and generate new multidisciplinary collaborations that will form the basis of new discoveries that we can discuss at the next BEM Summit.

## Introduction

Bioelectronic medicine (BEM) is evolving at a rapid and steady pace, bringing device-based therapies to the clinic that have the potential to treat human disease and improve health. With the potential of BEM to treat a wide range of disorders, a cross-disciplinary exchange of ideas and efforts is needed to optimize problem-solving and capitalize on current progress. The 2022 BEM Summit, held on October 11 and 12th, 2022, was an in-person meeting that brought together leaders in the fields of neuroscience, biomedical engineering, molecular biology, immunology, and technology. It was held at the Garden City Hotel, Garden City, New York and brought together more than 170 attendees from both academia and industry. The Summit was chaired by Dr. Yousef Al-Abed of the Feinstein Institutes for Medical Research and included more than 35 invited speakers, with keynote talks delivered by Dr. Kevin J. Tracey of the Feinstein Institutes for Medical Research and Dr. Warren M. Grill of Duke University.

To maximize potential opportunities for discussion and collaboration across the diverse set of attendees, the Summit was organized as a series nine scientific sessions over two days that were either open panel-type discussions with a Moderator, or traditional scientific sessions with slide-based presentations (Table 1). There was also a scientific Poster Session at the end of Day 1 that included a total of 39 abstract presentations. What follows in this Meeting Report is a summary of the scientific sessions and our takeaways from the 2022 BEM Summit.

**Table 1:** Full list of scientific sessions and speakers at the 2022 BEM Summit

**Session**	**Topic**	**Host, Moderator**	**Speakers, Panelists**
**Day 1**			
1	Neurotechnology in the treatment of brain diseases: Successes, challenges, and new frontiers	Host: Stavros Zanos, M.D. Ph.D Moderator: Robert Froemke, Ph.D.	Riki Banerjee, Ph.D., Tim Denison, Ph.D., Florian Solzbacher, Ph.D.
2	Bioelectronic medicine for neuropsychiatric disorders	Host and Moderator: Anil Malhotra, M.D.	Miklos Argyelan, M.D., Marom Bikson, Ph.D., Colleeen Hanlon, Ph.D., Daphne Voineskos, Ph.D.
3	Emerging devices and neural interfaces: Does innovation happen by addressing known gaps in technology or is it best driven by addressing researcher needs?	Host: Timir Datta-Chaudhuri, Ph.D Moderator: Eric Van Gieson, Ph.D.	Shadi Dayeh, Ph.D., Dimitrios Koutsouras, Ph.D., Flavia Vitale, Ph.D., Jesse Wheeler, Ph.D.
4	Decoding of neural signals and the use of data science and machine learning in neural systems as applied to bioelectronic medicine	Host and Moderator: Theodoros Zanos, Ph.D.	Konrad Koerding, Ph.D., Lorenzo Rossi, Ph.D., Maryam Shanechi, Ph.D.
5	Neural regulation of immunity: Controlling the immune response through the peripheral nervous system	Host and Moderator: Eric Chang, Ph.D.	Jeremy Borniger, Ph.D., Gloria Choi, Ph.D., Brian Kim, M.D., Asya Rolls, Ph.D.
	**End of Day 1, Poster Session**		
**Day 2**			
6	Bioelectronic approaches to the treatment of peripheral organ disorders	Host: Stavros Zanos, M.D. Ph.D Moderator: Eric Hudak, Ph.D.	Jeffrey Ardell, Ph.D., Dennis Bourbeau, Ph.D., Kevin Otto, Ph.D.
7	Neurotech: The journey from bench to bedside	Host and Moderator: JoJo Platt	Jennifer Ernst, M.B.A., Amy Kruse, Ph.D., Stephanie Lacour, Ph.D., Erika Ross, Ph.D.
8	Bioelectronic data in the wild: Using digital health data from consumer and clinical devices to inform biomarkers of disease	Host: Theodoros Zanos, Ph.D Moderator: Erika Ross, Ph.D.	Oliver Armitage, Ph.D., Siddarth Dani, M.S., Leah Muller, M.D. Ph.D., Brian Pepin, M.S.
9	Translating brain computer interfaces: Are we reaching an inflection point?	Host: Florian Solzbacher, Ph.D Moderator: Robert Gaunt, Ph.D.	Matt Angle, Ph.D., Jennifer Collinger, Ph.D., Robert Franklin, Ph.D.

## Scientific sessions

### Day 1

**Fig. 1:**
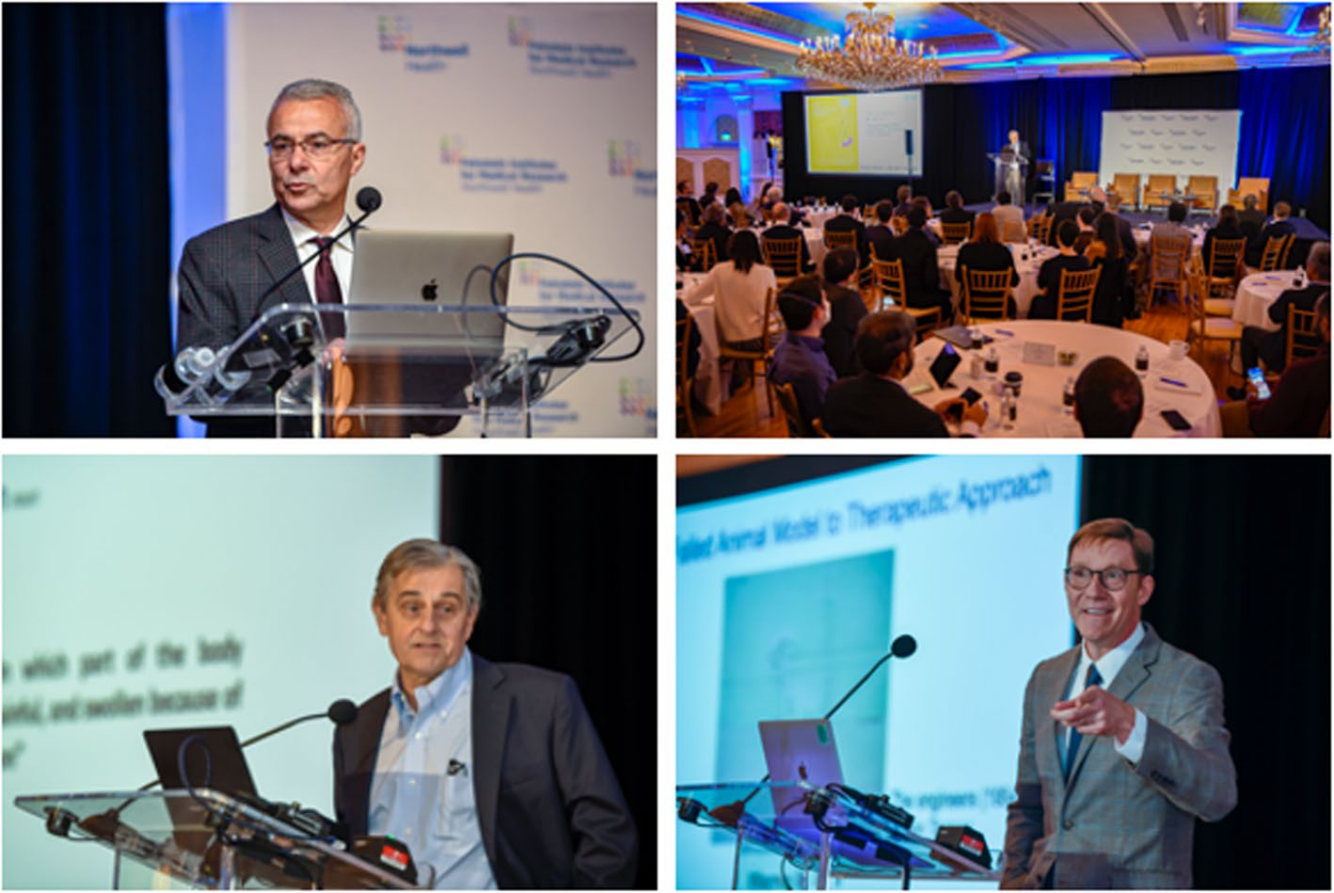
Photographs from the 2022 BEM Summit. Top left, BEM Summit Chair Dr. Yousef Al-Abed. Top right, The meeting room in Garden City Hotel, New York. Bottom left, Day 1 keynote speaker Dr. Kevin Tracey. Bottom right, Day 2 keynote speaker Dr. Warren Grill. Photo credits: Marc Farb, Sigma U.S.A.

Following opening remarks by the Meeting Chair Dr. Al-Abed, the meeting began with a keynote lecture by Dr. Kevin J. Tracey, President and C.E.O. of the Feinstein Institutes for Medical Research and Professor in the Institute of Bioelectronic Medicine (Fig. 1). Dr. Tracey spoke about inflammation as a primary cause of human disorders and mortality, stating that if we could find a way to control inflammation, then we could possibly extend the human lifespan by decades. This would be a transformational development in human history, and BEM may enable this possibility. Dr. Tracey went on to outline the discovery of *the inflammatory reflex* and its efferent arm – *the cholinergic anti-inflammatory pathway* and the scientific dogmas that were challenged by these findings (Barker and Billingham 1977; Tracey 2002, 2007). The dogmatic view is that the sympathetic and the parasympathetic parts of the autonomic nervous system function in opposition to regulate physiological function. However, recent work has identified a brainstem locus that inhibits the cytokine tumor necrosis factor, ultimately linking the parasympathetic part (i.e. vagus nerve) and a sympathetic nerve (i.e. splenic nerve) in the regulation of inflammation (Kressel et al. 2020). It is now clear that the vagus nerve controls immune cell function in the spleen through several neural circuits, including cholinergic signaling via the celiac ganglion (Pavlov et al. 2018; Rosas-Ballina et al. 2008). Work by another group identified that vagus nerve stimulation (VNS) activates distinct neuroimmune circuits converging in the spleen to protect mice from kidney injury, again confirming the direct interactions between the parasympathetic and sympathetic parts of the autonomic nervous system (Tanaka et al. 2021). Dr. Tracey also outlined work by Linda Watkins and colleagues that showed a vagotomy can block interleukin-1 (IL-1)-induced sickness behavior, identifying the vagus nerve as a pathway for immune sensory signals (Fleshner et al. 1995). Subsequent work from the Feinstein Institutes identified that there are in fact cytokine-specific sensory neural signals carried by the vagus nerve (Steinberg et al. 2016; Zanos et al. 2018). Recent work from the Tracey lab showed that transient potential receptor ankyrin 1 (TRPA1) channels are required for IL-1-mediated vagus nerve signaling to occur (Silverman et al. 2023). The identification of vagus nerve sensory receptors that are necessary for the inflammatory response supports the notion that the nervous and immune systems interact frequently during immune responses. Another major discovery in the neural regulation of inflammation is the role of the brain in the physiological response to inflammation and the identification of an “immunological homunculus” (Diamond and Tracey 2011), as shown by specific insular cortex neurons that have been demonstrated to encode and retrieve specific immune responses (Koren et al. 2021). Concluding his keynote, Dr. Tracey spoke about ongoing recent clinical trials with VNS that have shown the anti-inflammatory and disease-alleviating activity of this approach in patients with rheumatoid arthritis (Koopman et al. 2016). He ended with an inspiring statement for the future of BEM that today’s progress in immunosuppression can lead to tomorrow’s progress in immunoregulation. In other words, once we understand the molecular mechanisms and neural circuits regulating one aspect of physiology, we can begin to extend that capability into other domains.

Following Dr. Tracey’s keynote, the first scientific session (Session 1) was on “Neurotechnology in the treatment of diseases: Successes, challenges, and new frontiers”, which was hosted by Dr. Stavros Zanos from the Feinstein Institutes for Medical Research and moderated by Dr. Robert Froemke from New York University (Table 1). Speakers in this session included Dr. Riki Banerjee from Synchron, Dr. Florian Solzbacher from the University of Utah and Blackrock Neurotech, and Dr. Tim Denison from the University of Oxford. The speakers presented different aspects of bioelectronic therapies for treating brain disorders, all based on bidirectional communication between a medical device and the nervous system. Dr. Banerjee discussed Synchron’s “stentrode” device, which is an endovascular brain computer interface (BCI) device. She covered how the stentrode evolved from concept to preclinical tests, and then to recently initiated clinical trials in patients with paralysis (Mitchell et al. 2023, Oxley et al. 2016). Dr. Solzbacher discussed the widely used Utah array BCI, which allows the recording of neural signals and neurostimulation when implanted into the cortex. Bidirectional Utah arrays are currently being tested extensively in preclinical research and clinically in patients with paralysis and other neurological disorders (Ezzyat et al. 2018; Wendelken et al. 2017). Dr. Denison’s talk focused on the need for the alignment of device engineering with the clinical needs and daily lives of patients. One approach for this would be to couple neuromodulation with real-time monitoring of neural circuits in a closed-loop fashion. For example, by monitoring a Parkinson’s disease patient’s individual circadian rhythms, the deep brain stimulation (DBS) device could be switched off when the patient is resting (Cagan et al. 2019; Smyth et al. 2023). Closed-loop neurological devices respond to the dynamic nature of a patient’s needs and optimize the timing, and other parameters, of neurostimulation therapies. All the speakers emphasized the importance of collaboration between engineers, clinicians, and patients to create effective neurotechnology solutions. Overall, the session highlighted the successes, challenges, and new frontiers in the development of bioelectronic therapies for brain disorders.

The next session (Session 2) was titled, “Bioelectronic medicine for neuropsychiatric disorders”, which was hosted and moderated by Dr. Anil K. Malhotra of the Feinstein Institutes for Medical Research and Zucker Hillside Hospital. The speakers in this session included Dr. Marom Bikson from City University of New York, Dr. Colleen Hanlon from Wake Forest School of Medicine, Dr. Daphne Voineskos from the University of Toronto, and Dr. Miklos Argyelan from the Feinstein Institutes for Medical Research. Technologies using noninvasive brain stimulation in psychiatry were the focus of this session, particularly transcranial magnetic stimulation (TMS) and electroconvulsive therapy (ECT). Dr. Bikson presented work on how electrical brain stimulation modulates vascular function and explained the importance of neurovascular coupling to regulate brain function. Noninvasive brain stimulation methods such as transcranial direct current stimulation (tDCS), ECT, and TMS, are techniques that have been shown to modulate vascular function, so blood–brain barrier and capillary effects should be accounted for when these modalities are used (Bahr-Hosseini and Bikson, 2021; Khadka et al. 2023). Dr. Argyelan showed how volumetric brain differences could be related to cognitive side effects in ECT patients, and how electric field modeling can be used with in conjunction with structural magnetic resonance imaging (MRI) to minimize treatment-associated side effects (Argyelan et al. 2019; Argyelan et al. 2021). Dr. Hanlon shared data on the application of TMS in drug-cue reactivity among substance-dependent participants who used either cocaine, alcohol, or nicotine (Dunlop et al. 2017; Hanlon et al. 2017). Dr. Voineskos also covered TMS but with a focus on treatment-resistant depression. In this work, she coupled TMS with electroencephalography (EEG) to show that specific aspects of the EEG signals are linked to specific neurotransmitter receptors in the brain. Dr. Voineskos noted that variability in stimulation efficacy could be linked to anatomical variations in the brain folding patterns of each patient, again highlighting the importance of accounting for individual variance across human subjects when performing neuromodulation (Blumberger et al. 2021; Voineskos et al. 2021).

Session 3 was titled “Emerging devices and neural interfaces: Does innovation happen by addressing known gaps in technology or is it best driven by addressing researcher needs?”. The session was hosted by Dr. Timir Datta-Chaudhuri from the Feinstein Institutes for Medical Research and moderated by Dr. Eric Van Gieson, a former program manager at the Defense Advanced Research Projects Agency (DARPA). Speakers in this session included Dr. Flavia Vitale from the University of Pennsylvania, Dr. Shadi Dayeh from the University of California San Diego, Dr. Jesse Wheeler from Inner Cosmos, and Dr. Dimitrios Koutsouras from Interuniversity Microelectronics Centre (IMEC) Netherlands. Dr. Vitale spoke about using nanotechnology to address some of the materials challenges for bioelectronics. In particular, she reviewed a number of applications of MXenes, a new class of materials that offer excellent mechanical and electrical properties while being far easier to manufacture and scale-up than traditional microfabricated devices (Driscoll et al. 2021; Garg and Vitale 2023). Dr. Dayeh spoke about addressing unmet needs in clinical applications of tech-driven neurotechnology. He specifically addressed how to overcome challenges in electrode impedance in high-density thin-film electrode arrays, which allow previously unmet levels of spatiotemporal resolution in brain recording (Liu et al. 2017). Dr. Wheeler brought the unique perspective of a MedTech startup to the meeting, presenting a minimally invasive new BCI “digital pill” developed by Inner Cosmos to treat depression. Dr. Koutsouras presented an overview of IMEC’s strategic vision of applications of semiconductor technologies, and their specific focus on healthcare. He also discussed specific research projects on developing new methods for selective peripheral nerve stimulation, which fall along IMEC’s roadmap for electroceuticals technologies (He et al. 2022).

The next session (Session 4) remained on the topic of neural signals, entitled “Decoding of neural signals and the use of data science and machine learning in neural systems as applied to bioelectronic medicine”. This session was hosted and moderated by Dr. Theodoros Zanos from the Feinstein Institutes for Medical Research. Speakers in this session included Dr. Maryam Shanechi from the University of Southern California, Dr. Konrad Koerding from the University of Pennsylvania, and Dr. Lorenzo Rossi from Newronika. Dr. Shanechi presented extensive work from her lab on next-generation neurotechnology interfaces, applying engineering principles to the research and treatment of neurological and neuropsychiatric disorders. Specifically, she spoke about the opportunities of actively modeling neural signals across multiple brain regions, using deep learning techniques, that enable brain signal decoding to longitudinally track symptom levels of various conditions, such as depression (Sani et al. 2021). Dr. Koerding spoke about the tremendous opportunities and pitfalls of using novel machine learning algorithms to model neuronal circuits and decode brain signals, and specifically expanded on the use of auto-machine learning (auto-ML) approaches, which allows researchers to train and test multiple machine learning algorithms with a few lines of code. Dr. Koerding touched on the opportunity of democratization of modeling methodologies that these technologies bring, while reminding the audience of the importance of a clear understanding of the amount and quality of the data used, as well as the fundamentals of these approaches to avoid common pitfalls such as overfitting (Achakulvisut et al. 2021). Dr. Rossi spoke about Newronika’s efforts in developing a closed-loop system for deep brain stimulation (DBS). He described both the capabilities of the device to records brain signals during stimulation delivery from the same DBS electrodes, that include the digital signal processing steps enabling adjustments of stimulation parameters in real-time (Marceglia et al. 2022).

The final session of Day 1 (Session 5) transitioned to the field of neuroimmunology, specifically on the “Neural regulation of immunity: Controlling the immune response through the peripheral nervous system”. This session was hosted and moderated by Dr. Eric Chang of the Feinstein Institutes for Medical Research. Speakers in this session included Dr. Gloria Choi from the Massachusetts Institute of Technology, Dr. Asya Rolls from Technion Israel Institute of Technology, Dr. Brian Kim from the Icahn School of Medicine at Mount Sinai, and Dr. Jeremy Borniger from Cold Spring Harbor Laboratory. The talks in this session put a spotlight on neural circuits in the central nervous system (CNS) and peripheral nervous system (PNS) that regulate biology, physiology, and immunology. Dr. Rolls opened the session sharing data on how reward pathway signaling may mediate beneficial aspects of the placebo effect by driving symptom improvement through expectations. Activation of the ventral tegmental area (VTA), a key reward system in the brain, can boost innate and adaptive immune responses through a potential sympathetic nervous system pathway (Ben-Shaanan et al. 2016). She also reviewed recent findings from her lab on the role of insular cortex neurons in the encoding and reactivation of immune responses in colitis and peritonitis, highlighting the role of the brain in the representation of specific immune states (Koren et al. 2021; Rolls 2023). Dr. Choi presented results on maternal immune activation, which leads to autism-like social deficits in offspring and can be traced back to specific interneurons in the cortex. Specifically, she showed that T helper 17 cells and maternal interleukin-17 are linked to abnormal cortical development and deficits in social interactions (Choi et al. 2016; Reed et al. 2020). These results demonstrate how neuroimmune interactions during neurodevelopment can play a critical role in the manifestation of behavioral abnormalities. Dr. Kim discussed the molecular and neural mechanisms of chronic itch, in particular, how Janus kinase 1 (Jak1) and several interleukins play a role in atopic dermatitis and other skin itch conditions (Kim et al. 2020; Guttman-Yassky et al. 2023). He emphasized the intimate links between skin inflammation, sensory neurons, and primary cells of the immune system, including immunoglobulin E and B lymphocytes (Oetjen et al. 2017). Importantly, these basic science discoveries have led to new Jak-based drug therapies for treating patients with various dermatological conditions (Kim 2022). Dr. Borniger presented data on glucocorticoids as regulators of the immune system, connecting major hypothalamic regions of the brain (paraventricular nucleus and lateral hypothalamus) via corticotropin-releasing hormone neurons (Li et al. 2020). He also presented data on how cancers, specifically breast cancer, are known to affect homeostatic processes in the body and brain (Francis and Borniger 2021).

Following closing remarks from Chair Dr. Al-Abed, there was a well-attended poster session where 39 abstracts were presented [Abstracts from the Fifth BEM Summit, 2023], allowing for lively discussions with many of the early career researchers who are doing the hands-on work in the laboratory, clinics, and at the bench.

### Day 2

Following introductory remarks from Dr. Tracey, the second day of the Summit began with a keynote talk by Dr. Warren M. Grill, Distinguished Professor of Biomedical Engineering at Duke University (Fig. 1). Dr. Grill’s talk focused on the clinical disorder of bladder under-activity and the role of nervous system control, specifically the cholinergic system, in this vital physiological function. Patients and preclinical models with bladder under-activity have reduced voiding pressures and efficiency, which can be treated with neurostimulation of peripheral nerves (Gonzalez et al. 2021; Hokanson et al. 2021). Loss of control over voiding, for example in spinal cord injury, significantly impacts patient autonomy and quality of life. Dr. Grill also spoke about a range of neuromodulation approaches that can be used to treat a wide range of neurological disorders, including DBS for movement disorders and spinal cord stimulation for chronic pain. With other neuromodulation techniques, such as TMS, he noted that we should try to understand the cellular and network-level effects of noninvasive approaches to stimulate the brain. By developing novel methods to measure evoked potentials and by using biophysically-based computational models to estimate neural activation, we can improve our ability to selectively stimulate the nerve and brain areas that exert neural control of various physiological functions (Kent et al. 2015). Dr. Grill emphasized that the design of novel electrodes and stimulation waveforms for selective stimulation are critically important areas of development for current and future BEM therapies.

Following Dr. Grill’s keynote, the first session of the day (Session 6) was on “Bioelectronic approaches to the treatment of peripheral organ disorders”. The session was hosted by Dr. Stavros Zanos and moderated by Dr. Eric Hudak from the National Institutes of Health (NIH). Speakers in this session included Dr. Jeffrey Ardell from the University of California Los Angeles, Dr. Kevin Otto from the University of Florida, and Dr. Dennis Bourbeau from Case Western University. Dr. Ardell focused on the use of VNS in the treatment of heart failure and cardiac abnormalities. Through a detailed overview of the neural circuits that innervate the heart and the physiology of autonomic regulation, he provided evidence for why VNS is a rational treatment for heart failure, while acknowledging the past challenges of failed heart failure bioelectronic treatments (Konstam et al. 2022). Dr. Otto discussed his rationale for using autonomic nerve regulation for the treatment of diabetes. Specifically, he discussed preclinical work on recording and stimulating the autonomic nervous system to regulate glucose metabolism in animal models of diabetes (Dirr et al. 2020; Dirr et al. 2023). This work is currently being translated into clinically relevant therapies to treat diabetic patients. Dr. Bourbeau discussed an often underappreciated patient perspective, which is the process of developing novel bioelectronic therapies for patients with spinal cord injury (SCI) and disorders of bladder function as a result of their injury (Bourbeau et al. 2020). He provided real-life insights from SCI patients and emphasized the importance of engaging with patients at the early stages of developing and testing new neurostimulation devices to maximize therapeutic value and the likelihood of success. Overall, this session highlighted the potential of neurostimulation therapies to treat complex disorders of organs that lie outside the traditional definition of neurological disease.

The next session on this day (Session 7) was entitled “Neurotech: The journey from bench to bedside”, which was hosted and moderated by JoJo Platt from Platt and Associates. Speakers in this session included Dr. Stephanie Lacour from Ecole Polytechnique Fédérale de Lausanne (EPFL), Dr. Erika Ross from Abbott, Dr. Jennifer Ernst from Tivic Health, and Dr. Amy Kruse from Prime Movers Lab. In this session, the speakers discussed several different aspects of working, and leading, various neurotech companies. Dr. Ross spoke about the strength of companies that are “built to keep”, as opposed to being built with the intent of being ultimately sold or acquired. Dr. Kruse talked strategy about how to work with venture capital firms and how to plan for recruiting staff. She emphasized that venture capital is a relationship business, so potential entrepreneurs should try to engage with them through their white papers and other content that they share. Dr. Ernst discussed different strategies to obtain early angel investments and how starting a company requires a different skillset than running a company. She advised that people gain experience in smaller companies to learn how every part of a company runs, then to move to a larger company to see how processes operate at scale.

Session 8 was focused on “Bioelectronic data in the wild”, hosted by Dr. Theodoros Zanos from the Feinstein Institutes for Medical Research and moderated by Dr. Erika Ross from Abbott. Speakers in this session included Dr. Brian Pepin from Rune Labs, Dr. Oliver Armitage from BIOS Health, Dr. Siddharth Dani from Element Science, and Dr. Leah Muller from Saluda Medical. The speakers in this session discussed various emerging topics, including opportunities and pitfalls using real-world evidence/data and advanced data analytics, machine learning, and artificial intelligence in pre-market and post-market decisions. They also discussed the pros and cons of incorporating sensing capabilities in the device versus relying on external data, including electronic health records, registries, billing claims, wearables, and patient-generated/reported data. They spoke about the challenges and strategies using different physiological data modalities – from syncing, to data (sensor) quality/accuracy, to patient compliance, to multimodal data analysis methods. The speakers pointed out that the field is progressing from the age of initial algorithm development and data collection to “The battle of the algorithms”. As more bioelectronic data becomes available, there will be major challenges, both operational and regulatory, of deploying these algorithms in the wild.

The final scientific session (Session 9) of the meeting moved toward the translation of science into implantable devices. This session was titled, “Implantable brain computer interfaces: Are we reaching an inflection point?” and was hosted by Dr. Florian Solzbacher from Blackrock Neurotech and moderated by Dr. Robert Gaunt from the University of Pittsburgh. Panelists in this session included Dr. Matt Angle from Paradromics, Dr. Robert Franklin from Blackrock Neurotech, and Dr. Jennifer Collinger from the University of Pittsburgh. Dr. Solzbacher spoke about and detailed the numerous neural prosthetic devices that are currently in use to interface with the human brain and rely on Blackrock Utah array technology. He emphasized that patient feedback on the BCI strongly influences their device development strategies and that there could be improved logistics for time management between device timelines, patients, and regulatory agencies. Several panelists spoke about an ‘inflection point’ for BCIs and a potential plateau that is preventing BCIs from reaching more widespread adoption beyond the current levels. The ability to move past this plateau could depend on other non-scientific aspects, such as advocacy and public awareness. There was a general consensus that patient input was crucial for the prioritization of therapeutic goals.

As with any field of study, new and emerging talent should be elevated and supported whenever possible. As such, the 2022 BEM Summit concluded on Day 2 with platform oral presentations from the top three submitted poster Abstracts, as decided by a panel of judges. These platform presentations were delivered by: Fatima Alrashadan from Rice University on wireless power and closed-loop bioelectronics using magnetoelectric technology; Dr. Mihaly Hajos from Cognito Therapeutics on 40 Hz sensory stimulation to treat neurodegenerative diseases; Dr. Amparo Güemes González from the University of Cambridge on bioelectronic devices for vagus nerve recordings of metabolic information.

## Summary and future directions

Over the two-day BEM Summit, several common themes emerged from the scientific sessions and subsequent discussions. First, the importance of accounting for individual subject variability in biology and designing devices that can monitor these variances over time. Secondly, most of the speakers’ presentations underscored the importance of understanding the scientific underpinning of bioelectronic therapies and the neural or molecular mechanisms that mediate specific physiological functions. Finally, a major important point that should be kept top-of-mind for both academic and industry researchers is to ensure that patient perspectives are taken into account during the development and design of bioelectronic therapies.

A roadmap for the emerging field of BEM would be beneficial as there is such a diverse group of researchers in this space, ranging from material scientists to protein biologists. The 2022 BEM Summit was an opportune moment to bring together leaders in this field to share the latest advances from academic laboratories and industry. The sessions that included several neurotech startup companies were of particular interest to the next generation of researchers and entrepreneurs who might lead future companies aiming to treat disorders with bioelectronic approaches. It is our hope that a future BEM Summit and similar BEM conferences will help researchers around the world to capitalize and collaborate on emerging bioelectronic therapies and technologies to treat human disease.

## Data Availability

Not applicable.

## Abbreviations

BCI: Brain computer interface
BEM: Bioelectronic medicine
CNS: Central nervous system
DARPA: Defense Advanced Research Projects Agency
DBS: Deep brain stimulation
ECT: Electroconvulsive therapy
EEG: Electroencephalogram
EPFL: Ecole Polytechnique Fédérale de Lausanne
IL-1: Interleukin-1
IMEC: Interuniversity Microelectronics Centre
ML: Machine learning
NIH: National Institutes of Health
PNS: Peripheral nervous system
SCI: Spinal cord injury
tDCS: Transcranial direct current stimulation
TMS: Transcranial magnetic stimulation
TRPA1: Transient receptor potential ankyrin-1
VNS: Vagus nerve stimulation
VTA: Ventral tegmental area
